# Emerging role of the KCNT1 Slack channel in intellectual disability

**DOI:** 10.3389/fncel.2014.00209

**Published:** 2014-07-28

**Authors:** Grace E. Kim, Leonard K. Kaczmarek

**Affiliations:** Departments of Pharmacology and Cellular & Molecular Physiology, Yale University School of MedicineNew Haven, CT, USA

**Keywords:** Slack, KCNT1, intellectual disability, Fragile X syndrome, epilepsy

## Abstract

The sodium-activated potassium K_Na_ channels Slack and Slick are encoded by *KCNT1* and *KCNT2*, respectively. These channels are found in neurons throughout the brain, and are responsible for a delayed outward current termed *I*_KNa_. These currents integrate into shaping neuronal excitability, as well as adaptation in response to maintained stimulation. Abnormal Slack channel activity may play a role in Fragile X syndrome, the most common cause for intellectual disability and inherited autism. Slack channels interact directly with the fragile X mental retardation protein (FMRP) and *I*_KNa_ is reduced in animal models of Fragile X syndrome that lack FMRP. Human Slack mutations that alter channel activity can also lead to intellectual disability, as has been found for several childhood epileptic disorders. Ongoing research is elucidating the relationship between mutant Slack channel activity, development of early onset epilepsies and intellectual impairment. This review describes the emerging role of Slack channels in intellectual disability, coupled with an overview of the physiological role of neuronal *I*_KNa_ currents.

## INTRODUCTION

An influx of sodium ions through sodium channels or neurotransmitter receptors triggers a sodium-sensitive potassium current (*I*_KNa_), which is found in a diverse range of neuronal cell types. In many cases, *I*_KNa_ is mediated by the phylogenetically related K_Na_ channel subunits Slack and Slick (Bhattacharjee and Kaczmarek, 2005). Where Slack or Slick is expressed, *I*_KNa_ contributes to a late afterhyperpolarization that follows repetitive firing. *I*_KNa_ also regulates neuronal excitability and the rate of adaptation in response to repeated stimulation at high frequencies. Alterations in *I*_KNa_ have pathophysiological consequences, as suggested by reports of human mutations found in the Slack-encoding gene *KCNT1* (Barcia et al., 2012; Heron et al., 2012; Martin et al., 2014). Slack channels are hence associated with several early onset epileptic encephalopathies. Epilepsies associated with each one of the Slack mutations are in turn associated with a severe delay in cognitive development. Importantly, these new findings strengthened an earlier connection between Slack channels and Fragile X syndrome (FXS); Slack channels interact with FMRP (Fragile X Mental Retardation protein; Brown et al., 2010), which is absent in FXS patients. FXS as a condition is also associated with an increased incidence of childhood seizures, and is the most commonly inherited form of intellectual disability and autism. These observations suggest that Slack channels are developmentally important modulators of cell plasticity underlying normal cognitive development.

This review summarizes studies that have focused on the physiological and pathophysiological role of *I*_KNa_, with a particular focus on Slack channels, and also discusses implications for future research. The review is divided into the following parts. First, we describe the properties of Slack channels and physiological functions of the *I*_KNa_ current, drawing from both historical and more recent studies. Next, we compare and contrast some of the features of FXS and three epileptic encephalopathies (malignant migrating partial seizures of infancy, MMPSI; autosomal dominant nocturnal frontal lobe epilepsy, ADNFLE; and Ohtahara syndrome, OS; Barcia et al., 2012; Heron et al., 2012; Martin et al., 2014) that can result from mutations in Slack channels. In the last section, we cover the mechanisms by which Slack channel activity is altered in these conditions. In particular, we focus on the extent to which the development of intellectual disability can be attributed to the occurrence of the seizures themselves vs. alterations in cellular signaling pathways likely to be disrupted by Slack mutations.

## PROPERTIES OF KCNT1 SLACK CHANNELS

**FIGURE 1:**
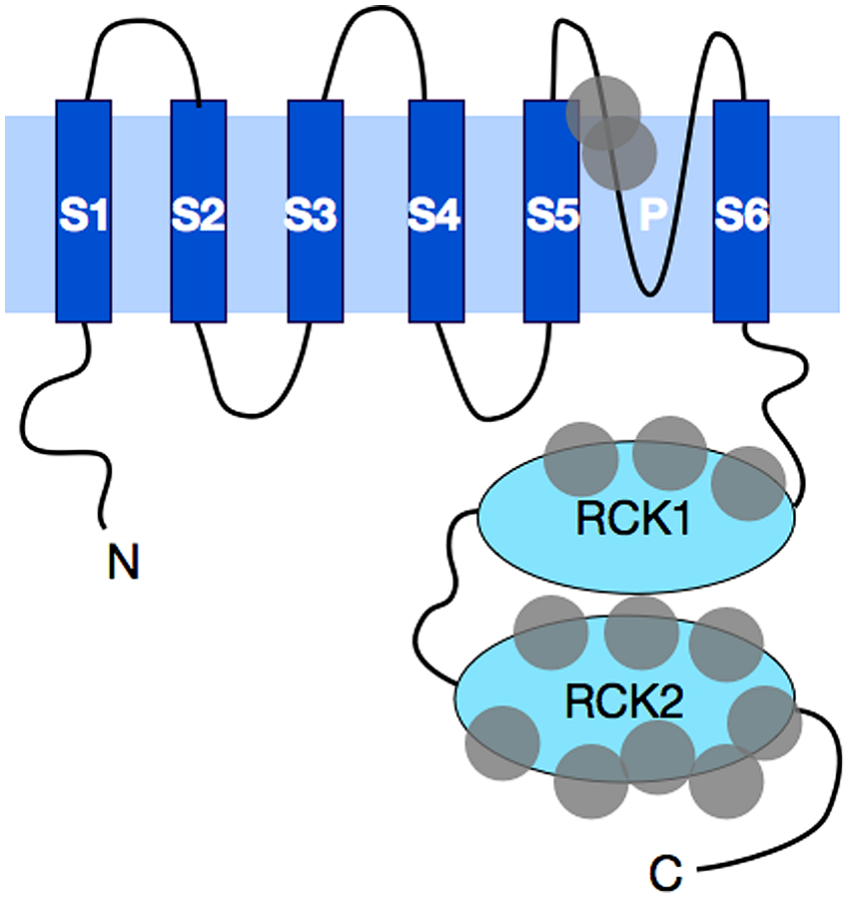
**A schematic diagram of Slack subunit topography.** Slack subunits have six transmembrane domains. These hydrophobic transmembrane segments are labeled as S1–S6, with the pore region between S5 and S6 indicated with the letter P. Four of these subunits assemble into a functional channel. Both the N- and C-terminal ends are cytosolic in Slack, with C-terminus being one of the longest found among all potassium channels. The C-terminus contains two RCK (regulators of K^+^ conductance) domains that stack on top of each other and form a gating ring underneath the channel opening pore. Gray circles represent the general locations where human mutations have been found. A total of thirteen distinct mutations have been found to date, and these mutations are discussed further in the text.

The *KCNT1* gene encodes the sodium-activated potassium channel called Slack (named for Sequence like a calcium-activated K^+^ channel). Slack channels resemble the well-known voltage-gated Kv channels in their topography and assembly. Like the Kv channels, Slack subunits have six hydrophobic, transmembrane segments (S1–S6) along with a pore-lining loop that is found between S5 and S6 (**Figure 1**). These subunits assemble as tetramers to form a functional channel that is voltage-dependent (Joiner et al., 1998). However, unlike the Kv family of channels, which use a set of positively charged residues along the S4 segment to sense changes in transmembrane voltage (Aggarwal and MacKinnon, 1996; Seoh et al., 1996), Slack channels have no charged residues in S4, and the corresponding mechanism for voltage-sensing in Slack channels is not yet understood. Another distinguishing feature of Slack channels is their very large cytoplasmic C-terminal domain, which is over 900 amino acids in length (Joiner et al., 1998), making Slack channels the largest know potassium channel subunits. In comparison, the C-terminal domain of one of the longest Kv family *eag* channels is only ∼650 amino acids in length (Warmke and Ganetzky, 1994).

The unitary conductance of the Slack channels expressed in heterologous systems ranges from 88 to 180 pS in symmetrical potassium solutions (Yuan et al., 2003; Chen et al., 2009; Zhang et al., 2012), while single channel conductances measured for Na^+^-activated K^+^ channels in native neurons range from 122 to 198 pS (Yang et al., 2007; Tamsett et al., 2009). At least three observations can explain the wide range and difference in the two expression systems. One confounding factor in measuring channel conductance is that both native *I*_KNa_ channels and Slack channels in expression systems are known to have multiple subconductance states, which in patch clamp experiments appear as brief, flickering short steps alternating with time spent in the fully open or the closed state (Yuan et al., 2003; Brown et al., 2008). We will revisit this particular property of Slack channels in our discussion of the pathophysiological consequences of aberrant changes in Slack channel activity. Secondly, diversity in the properties of native *I*_KNa_ can stem from the existence of multiple splice isoforms of Slack channels (Brown et al., 2008), and the fact that some Slack isoforms can form heteromers with related channel subunits such as Slick subunits (Joiner et al., 1998; Yang et al., 2007; Chen et al., 2009). Encoded by *KCNT2*, Slick subunits are distinct in their channel kinetic behavior and unitary conductances (Bhattacharjee et al., 2003). Heteromeric Slack/Slick channels also have properties that are yet different from those of either subunits expressed alone, and their response to modulation by protein kinases also differs from that of the homomeric channels (Chen et al., 2009). Evidence that Slack and Slick channels are co-expressed has been provided in auditory brainstem neurons, olfactory bulb and a number of other neurons (Bhattacharjee et al., 2005; Chen et al., 2009). Finally, in addition to the Slack and Slick channels, which are phylogenetically related (Salkoff et al., 2006), the evolutionarily more distant K_ir_3 inward rectifier potassium channels are also sensitive to cytoplasmic sodium ions, further increasing the diversity of native *I*_KNa_ channels. (Petit-Jacques et al., 1999).

## SLACK ENTERS INTO PROTEIN–PROTEIN INTERACTIONS WITH OTHER MEMBRANE PROTEINS AND CYTOPLASMIC SIGNALING PROTEINS

The Slack channel subunit interacts directly with the mRNA-binding protein FMRP, which regulates the probability of Slack channel opening (Brown et al., 2010; Zhang et al., 2012). Evidence for direct Slack channel-FMRP binding was first found in a yeast-two-hybrid assay, and confirmed by co-immunoprecipitation from synaptosomal lysates isolated from mouse brainstem and olfactory bulbs (Brown et al., 2010). This interaction appeared to be evolutionarily conserved, as the same finding was demonstrated in large bag cell neurons of the marine mollusk *Aplysia californica* (Zhang et al., 2012). Moreover, messenger RNA targets of FMRP can be co-immunoprecipitated with Slack from wild type mice but not from the *fmr^-/y^* mice lacking FMRP (Brown et al., 2010). Addition of an N-terminal fragment of FMRP (FMRP 1–298) that retains the majority of the known FMRP protein–protein interaction domains, but lacks the major mRNA binding sites to Slack channels in excised inside-out patches substantially increased channel mean open time (Brown et al., 2010). In part, this increase in Slack channel activity occurs by eliminating subconductance states and favoring openings to the fully open state.

Slack channel subunits also interact directly with TMEM16C (ANO3), a transmembrane protein found in non-peptidergic nociceptive neurons (Huang et al., 2013). Though closely related to the Ca^2+^-activated Cl^-^ channels TMEM16A and B, TMEM16C itself alone does not appear to function as an ion channel. Slack and TMEM16C can exist together in a protein complex and are colocalized in nociceptive neurons. Similar to FMRP, the presence of TMEM16C substantially increases the activity of Slack channels. Further discussion of the biological role of this interaction in nociceptive neurons is provided later in this review, but for now we turn to discuss neuronal cell types that express K_Na_ channels.

## LOCALIZATION OF SLACK AND SLICK SUBUNITS

Cloning of the K_Na_ Slack and Slick genes, *KCNT1* and *KCNT2*, and the development of specific antibodies have enabled a detailed study of their expression in the brain (Bhattacharjee et al., 2003, 2005; Yuan et al., 2003). These studies have confirmed that highest levels of Slack and Slick channels are found in the brain, with detection of lower levels in the heart and the kidney (Joiner et al., 1998; Yuan et al., 2003; Bhattacharjee et al., 2005; Brown et al., 2008). *In situ* hybridization and immunohistochemistry were systematically performed in the adult rat brain, and demonstrated that Slack transcripts and protein are abundantly expressed in neurons throughout all regions of the brain, including the brainstem, cerebellum, frontal cortex and the hippocampus (Bhattacharjee et al., 2002; Santi et al., 2006; Brown et al., 2008). Similar results are also reported in the mouse brain, where abundant mRNA expression has been found in the brainstem and the olfactory bulb (Brown et al., 2008).

## PHYSIOLOGICAL FUNCTIONS OF THE SLACK CHANNEL

### *I*_**KNa**_ IS A MAJOR COMPONENT OF THE DELAYED OUTWARD CURRENT IN NEURONS

The term *I*_KNa_ was first coined by Bader et al. (1985), who described in avian neurons an outward K^+^ current with dependence on [Na^+^]_i_ (**Table 1**). An independent study concurrently described similar currents in neurons isolated from the Crayfish (Hartung, 1985). In both studies, researchers observed changes in the outward K^+^ current in the presence and absence of the Na^+^ channel inhibitor tetrodotoxin (TTX), and concluded that a component of the neuronal outward current was sensitive to Na^+^ influx. Similar reports soon followed in a number of neuronal cell types, which led to the recognition of a previously unrecognized outward current that was sensitive to Na^+^ influx (Haimann et al., 1990; Dryer, 1991; Bischoff et al., 1998). A partial list of such cell types includes medial nucleus of the trapezoid body (MNTB), trigeminal, mitral, vestibular, and dorsal root ganglion (DRG) nociceptive neurons. Importantly, this list demonstrates that Slack channels are involved in the olfactory, auditory, vestibular and pain-sensing systems, all of which are critical to normal development and learning. For a more comprehensive review of Slack channel expression patterns, the reader is advised to Refs. (Bhattacharjee and Kaczmarek, 2005; Kaczmarek, 2013).

**Table 1:** Physiological role of Slack-mediated *I*_**KNa**_ in specific neuronal cell types (selected references).

Neuronal type	Animal/Age	*I*_KNa_ contribution	Reference
Ciliary and trigeminal ganglia	E7–8, chick or quail embryos	Sodium-dependent outward current	Bader et al. (1985)
Layer V neurons of sensorimotor cortex	Cats	sAHP, cellular excitability	Schwindt et al. (1989)
Spinal cord neurons	Lamprey (*Lampetra fluviatilis*)	sAHP, neuronal frequency regulation	Wallen et al. (2007)
MNTB principal neurons	P11, 129SV/EMS mice	Accuracy of timing of APs to high-frequency stimulation	Yang et al. (2007)
Olfactory bulb tufted/mitral cells, corpus striatum medium spiny neuron (MSN)	P2, rat pups	Delayed outward current	Budelli et al. (2009)
Olfactory bulb mitral cells	<1 m, C57BL/6 Kv1.3^-/-^ mice	Compensatory increase in Slack expression in Kv1.3^-/-^ mitral cells, leading to increased delayed outward current	Lu et al. (2010)
Peptidergic nociceptors in dorsal root ganglion (DRG)	E15, Sprague-Dawley rat embryos	APD, firing accommodation to stimulus, cellular excitability	Nuwer et al. (2010)
Non-peptidergic nociceptors in DRG	<1 m, F344 rats	APD, firing threshold, cellular excitability	Huang et al. (2013)
Vestibular afferent neurons (VAN)	<1 m, Sprague-Dawley rats	APD, rate of repolarization, amplitude of AHP, phase locking of APs	Cervantes et al. (2013)

*MNTB, medial nucleus of the tripezoid body; DRG, dorsal root ganglion; E, embryonic; P, post-natal; m, month; sAHP, slow afterhyperpolarization; AP, action potential; APD, action potential duration.*

### SLACK CHANNEL SUBUNITS ARE REQUIRED FOR *I*_KNa_

That Slack channel subunits contribute to *I*_KNa_ currents was demonstrated in later studies, using neonatal neurons isolated from the rat olfactory bulb, as well as in corpus striatum (Budelli et al., 2009; Lu et al., 2010). A component of the outward current similar to the *I*_KNa_ reported in the earlier studies was suppressed upon knocking-down Slack expression using the siRNA technique (Budelli et al., 2009). These studies contributed the surprising discovery that *I*_KNa_ represents a very major fraction of the total outward current of these neurons.

Levels of *I*_KNa_ channels are particularly high in mitral cells of the olfactory bulb (Egan et al., 1992; Bhattacharjee et al., 2002), in which the other major component of K^+^ current is carried by the voltage-dependent potassium channel subunit Kv1.3 (Kues and Wunder, 1992). The activity of Kv1.3 channels helps determine the firing patterns of mitral cells in response to odorant stimulation and/or glucose presence (Tucker et al., 2013). A very interesting phenotype results when Kv1.3 channels are deleted by homologous recombination in mice (Fadool et al., 2004). Levels of both *I*_KNa_ current and of Slack channel protein expression are substantially increased in Kv1.3^-/-^ mice (Lu et al., 2010). This *I*_KNa_ could be directly attributed to the Slack subunits by again knocking down Slack subunits with the siRNA technique, which suppressed the *I*_KNa_ currents (Lu et al., 2010). Loss of Kv1.3 channels, together with the upregulation of *I*_KNa_ currents, altered the kinetics of inactivation of K^+^ currents in the mitral cells, resulting in a decrease in action potential height and an increased adaptation of action potential firing in response to maintained stimulation (Fadool et al., 2004). Remarkably, these changes were associated with the development of increased numbers of olfactory glomeruli in the olfactory bulb and a 10,000-fold increase in the sensitivity of the Kv1.3^-/-^ mice to odorant stimuli.

### CONTRIBUTION OF *I*_KNa_ TO NEURONAL FIRING PATTERNS: REGULATION OF ADAPTATION TO MAINTAINED STIMULATION

In many neurons, *I*_KNa_ currents contribute to a long-lasting slow afterhyperpolarization (sAHP), which results from a slowly developing outward current evoked during sustained stimulation (Vergara et al., 1998). The period of reduced excitability afforded by sAHP is thought to protect the cell from repetitive, tetanic activity, and has been studied in layer V neurons of the sensorimotor cortex of the cat (Schwindt et al., 1988a,b). It has been shown that whereas the early part of the sAHP is dependent on Ca^2+^ influx during stimulation, the late part is Na^+^-sensitive. Furthermore, this late component of the sAHP is sufficient to reduce cellular excitability in the cat sensorimotor cortex layer V neurons (Schwindt et al., 1989). Performing slice recordings in the absence of Ca^2+^, Schwindt et al. (1989) showed that neuronal firing rate is attenuated for many tens of seconds following stimulation, matching the duration of Na^+^-dependent sAHP.

Similar Na^+^-dependent sAHPs have also been observed in a number of other neurons, including hippocampal pyramidal cells (Gustafsson and Wigstrom, 1983) and spinal cord neurons (Wallen et al., 2007). In motor neurons from the lamprey spinal cord, stimulation of action potentials at increasingly higher rates (from 2 to 8 Hz) progressively prolongs the time it takes for the membrane potential to return to baseline, an effect that can be attributed to the duration of the evoked sAHP. At lower firing rates, the Ca^2+^-sensitive early phase of the sAHP dominates the rate of recovery to the resting state. However, the contribution of the late *I*_KNa_-dependent phase of the sAHP to this effect becomes more significant with increasing firing frequencies. It appears then that the *I*_KNa_-mediated sAHP is likely to be a physiological modulator of neuronal excitability during rapid firing (Wallen et al., 2007).

### ROLE OF SLACK CHANNELS IN NOCICEPTION

Two studies focusing on the pain-sensing DRG nociceptors have shed further light on the role of *I*_KNa_ in neuronal excitability (Nuwer et al., 2010; Huang et al., 2013). In one study, siRNA-mediated technology was utilized to knock down Slack channels in the embryonic rat peptidergic nociceptors, demonstrating that these Slack-knockdown neurons were hyperexcitable compared to control neurons (Nuwer et al., 2010). The second study showed that the voltage threshold for action potential generation is significantly reduced in nociceptive neurons isolated from a TMEM16C^-/-^ rat (Gadotti and Zamponi, 2013; Huang et al., 2013). As was described earlier in this review, TMEM16C is a transmembrane protein found in non-peptidergic nociceptive neurons that binds Slack channel subunits and increases their channel activity. Consistent with this, the neurons from TMEM16C^-/-^ rats had reduced *I*_KNa_ currents. The TMEM16C^-/-^ rats also had increased thermal and mechanical sensitivity, as revealed in behavioral studies. That this increased sensitivity could be directly attributed to the change in Slack *I*_KNa_ current was confirmed by an *in vivo* Slack knockdown experiment in animals, which induced the same pattern of heightened sensitivities (Huang et al., 2013).

### ROLE OF SLACK CHANNELS IN TEMPORAL ACCURACY OF ACTION POTENTIAL FIRING

Slack/Slick channels are also expressed in high abundance in neurons of the MNTB within the auditory brainstem (Bhattacharjee et al., 2002; Yang et al., 2007). These neurons are capable of firing at rates up to ∼800 Hz with high temporal accuracy, a feature that is required for accurate determination of the location of sounds in space. Current clamp and voltage clamp experiments have demonstrated that activation of *I*_KNa_ currents increases temporal accuracy in these neurons at high rates of stimulation, in large part by increasing the membrane conductance close to the threshold for action potential generation (Yang et al., 2007). This reduces the time constant of the membrane and allows the timing of action potentials to be closely matched to the pattern of incoming stimuli. Pharmacological activation of Slack channels in these neurons has been shown to further increase timing accuracy in these cells, a finding that is consistent with numerical simulations of the firing patterns of these cells with and without *I*_KNa_ currents (Yang et al., 2007).

*I*_KNa_ currents also shape the neuronal firing in the vestibular system, which consists of four vestibular nuclei that receive input from the vestibular afferent neurons (Fitzpatrick and Day, 2004). The afferent neurons transmit information about head movements to help the organism stabilize gaze and maintain proper balance. Vestibular afferent neurons have characteristic resting discharge rates that adapt upon detecting angular and linear accelerations, thereby relaying vestibular information (Grillner et al., 1995). Cervantes et al. (2013) characterized *I*_KNa_ currents in rat vestibular ganglion neurons, and found that *I*_KNa_ currents regulate the phase-locking of action potential firing to a stimulus, as well as the firing regularity and discharge patterns of these neurons.

The summarized studies have demonstrated that *I*_KNa_ currents are a major physiological component of the outward current in neurons, where these currents help regulate intrinsic electrical excitability, as well as the manner in which neurons respond to patterns of incoming stimulation. These studies have led Budelli et al. (2009) to conclude “*in clinical and pharmacological studies, this previously unseen current system that is active during normal physiology represents a new and promising pharmacological target for drugs dealing with seizure and psychotropic disorders*,” an early prediction that would be realized by the finding of human mutations in Slack channels.

## SLACK CHANNELS IN COGNITIVE DISORDERS

Given that Slack channels appear as modulators of neuronal excitability and of neuronal adaptation to stimulation in a wide range of species, it is not surprising that alterations in Slack channel activity may have significant pathophysiological consequences. Furthermore, what is known about these pathologies strongly suggests that Slack channel activity is a critical component that ensures normal cognitive development. The finding that Slack channel activity is increased by direct complex formation with FMRP, the RNA-binding protein that is deleted in FXS, implicates Slack channel function in this syndrome (Brown et al., 2010). More specifically, there may be a clinically significant relationship between Slack channel activity and development of intellectual disability in FXS. Increasing evidence supports this hypothesis: epilepsy patients who have profound intellectual disability carry mutations in the Slack-encoding *KCNT1*. More than a dozen different *KCNT1* mutations have now been reported in the literature, in connection with three different types of seizures that occur in infancy or childhood, MMPSI, ADNFLE, and OS (Barcia et al., 2012; Heron et al., 2012; Martin et al., 2014). These findings strongly indicate a pathophysiological role for Slack channels in the abnormal development of intellectual function.

### INTELLECTUAL DISABILITIES

Seizures can have variations in onset and frequency, and may occur during childhood with little or no intellectual impairment [Engel and International League Against Epilepsy (ILAE), 2001]. A case in point is ADNFLE, epilepsy that can be caused by mutations either in the α-4, α-2 or β-2 subunits of the neuronal nicotinic acetylcholine receptor, encoded by the *CHRNA4*, *CHRNA2,* or *CHRNB2* genes respectively, or by mutations in the Slack channel. Severe intellectual disability, however, only occurs in those patients who carry Slack mutations (Heron et al., 2012). This implies that the seizure episodes themselves are unlikely to be the prime determinant of intellectual function. Intellectual disability is a salient feature in all patients diagnosed with FXS, and in some patients with epilepsy and/or autism spectrum disorder (ASD). Below, we explore the overlap in clinical manifestation among these three types of patient groups.

**FIGURE 2:**
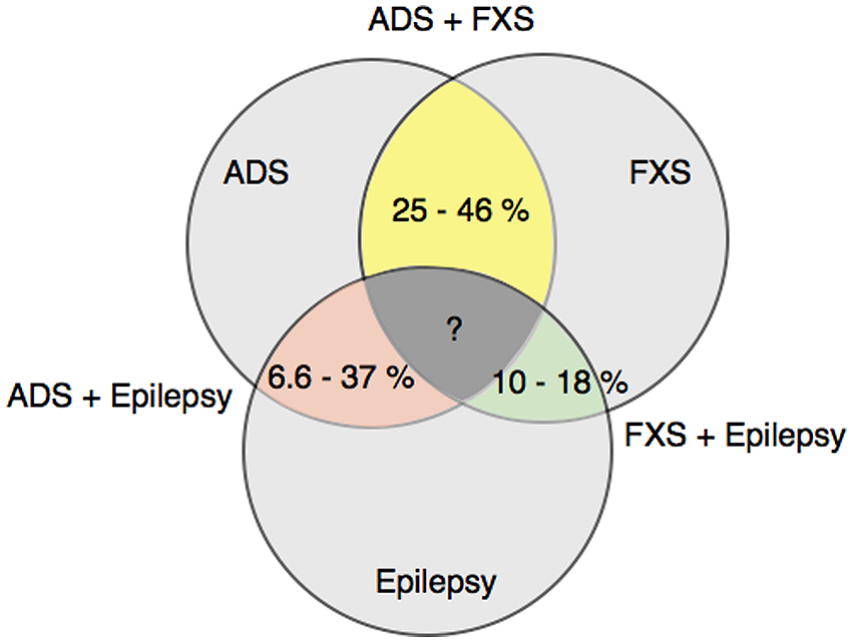
**Co-diagnosis rates among FXS, ASD and epilepsy patients.** This diagram shows the prevalence of co-diagnosis in fragile x syndrome (FXS), autism spectrum disorder (ASD) and epilepsy patients, as reported in Refs. (Musumeci et al., 1999; Muhle et al., 2004; Abrahams and Geschwind, 2008; Amiet et al., 2008; Bailey et al., 2008; Hernandez et al., 2009; Yasuhara, 2010; Jokiranta et al., 2014).

Fragile X syndrome, childhood epilepsies and ASD are notable for their heterogeneity of clinical manifestations in the behavioral and cognitive domains. Different combinations of these three disorders have also occurred together in patients. Numerous studies have reported a range of percentages for the prevalence of such overlapping patient groups, and are shown in **Figure 2**. The co-diagnosis rate of an ASD disorder in male Fragile X patients ranges from 25 to 46% (Muhle et al., 2004; Abrahams and Geschwind, 2008; Bailey et al., 2008; Hernandez et al., 2009). The corresponding rate for epilepsy in male Fragile X patients is lower, ranging from 10 to 18% (Musumeci et al., 1999; Muhle et al., 2004; Bailey et al., 2008), whereas the occurrence of epilepsy in ASD patients varies more widely from 6.6 to 37% (Amiet et al., 2008; Yasuhara, 2010; Jokiranta et al., 2014). Such a wide variation likely reflects methodological differences, as well as heterogeneity in sample population and etiology of the diseases. Even so, these studies are helpful in demonstrating the overlap among FXS, childhood epilepsy and ASD at the clinical diagnostic level. More pertinent to our discussion in this review, these findings raise the possibility that there could be a molecular link that controls intellectual disability development in each of the three clinical diseases.

### FRAGILE X SYNDROME

Fragile X syndrome is caused by functional absence of the FMRP, which usually arises due to hypermethylation and subsequent silencing of its gene *fmr1*, found on the X chromosome (Pieretti et al., 1991). FMRP is highly expressed throughout the brain in neurons, where it is found in both pre- and post-synaptic processes (Christie et al., 2009). One well-characterized function of this RNA-binding protein is to suppress the translation of these target mRNAs. Through mechanisms that are not fully understood, neuronal activity can release the suppression of some mRNAs (Khandjian et al., 2004; Stefani et al., 2004), leading to an activity-dependent increase in protein synthesis in synaptosomal regions (Bassell and Warren, 2008). FMRP binds to polyribosomes and specific mRNAs in neuronal dendrites, leading to the concept that it regulates local translation at these sites. FMRP is required for a number of forms of synaptic plasticity including mGluR1-mediated long-term depression (LTD; Li et al., 2002).

As described earlier, FMRP can also form complexes with Slack channel protein (Brown et al., 2010; Zhang et al., 2012), and in this manner directly regulate Slack channel activity. *I*_KNa_ currents were compared in MNTB neurons recorded in brain slices from the FMRP-deficient *Fmr1*^-/y^ mice vs. those from wild-type mice. As expected, outward *I*_KNa_ currents were smaller in *Fmr1*^-/y^ MNTB neurons, even though Slack subunit levels are not decreased (Brown et al., 2010). Conversely, increases in levels of FMRP can enhance *I*_KNa_ currents. This was demonstrated by the finding that introduction of the FMRP N-terminal 1–298 fragment into bag cell neurons of *Aplysia* increases *I*_KNa_ currents and hyperpolarizes the resting membrane potential (Zhang et al., 2012). These findings suggest a more versatile role for FMRP in both the presynaptic and postsynaptic elements of neurons, in addition to its function in the suppression of translation.

Slack is not the only ion channel that can interact with FMRP. Both the large-conductance calcium-activated BK potassium channel and Ca_V_2.2 voltage-dependent calcium channel have recently been shown to interact directly with FMRP, and these interactions regulate action potential width and neurotransmitter release (Deng et al., 2013; Ferron et al., 2014). It is possible that the activation of ion channels that are linked to FMRP serves as a local mechanism to regulate the translation of neuronal mRNAs (Zhang et al., 2012). These new findings collectively suggest that dysregulation of an acute modulation of neuronal excitability and transmission by FMRP may contribute to the intellectual disability associated with FXS.

### EPILEPSY

Epilepsy is estimated to affect 50 million people worldwide (World Health Organization, 2012). While seizures, presenting as abnormal patterns of synchronous activity in EEG recordings, can occur in isolation in both children and adults, these are distinguished from epilepsy, in which such abnormal activity is recurrent, and which may have an enduring clinical impact. The impact can manifest as neurobiological, cognitive, psychological, and/or social changes (Fisher et al., 2005). Although over 30 different kinds of epileptic seizures, or syndromes, are recognized as of 2013, each syndrome has considerable variation in etiology and health outcome [Engel and International League Against Epilepsy (ILAE), 2001; Berg et al., 2010].

Some epilepsies are channelopathies, and human mutations in a number of genes encoding ligand-gated receptors or ion channels have been found in epilepsy patients (Steinlein et al., 1995; Scheffer and Berkovic, 1997; Charlier et al., 1998; Zuberi et al., 1999; Escayg et al., 2000; Brenner et al., 2005). The advancement and wider use of sequencing technologies such as whole exome sequencing, which can identify *de novo* mutations in single probands, are reshaping the genomics approach to understanding epileptogenesis, and rapidly expanding the list of proteins mutated in epilepsy patients. In this next section, we consider in particular the three types of seizures associated with mutations in the Slack-encoding *KCNT1* gene, accompanied by a summary table of selected clinical reports from the literature.

### MALIGNANT MIGRATING PARTIAL SEIZURES IN INFANCY

Malignant migrating partial seizures in infancy was first described by Coppola et al. (1995), as a new distinct early onset (<6 months) seizure type with a characteristic random pattern of electrical discharges recorded on the brain electrical encephalogram (EEG). Since then, numerous other groups have also identified patients who fit these original criteria, selected references of which are reviewed and summarized in **Table 2** (Coppola et al., 1995, 2006, 2007; Okuda et al., 2000; Veneselli et al., 2001; Gross-Tsur et al., 2004; Marsh et al., 2005; Hmaimess et al., 2006; Caraballo et al., 2008; Carranza Rojo et al., 2011; Sharma et al., 2011; Barcia et al., 2012; Lee et al., 2012; Ishii et al., 2013; McTague et al., 2013; Milh et al., 2013). Ongoing analyses of these patients using EEGs, brain imaging and DNA sequence analysis continue to shape the field’s understanding of this focal seizure in infancy.

**Table 2:** Summary of clinical reports on MMPSI patients.

No. of patients reviewed	96
No. of Refs. Reviewed	16
Males	42.5%
**Age at onset**
Average	7.6 week
Range	1 day – 7 month
**Prognosis**
Developmental delay	71%
Normal development	1%
Hypotonia	56%
Deceased	21%
**Causes – brain abnormalities**
Normal	16
Atrophy	24
Delayed myelination	12
Encephalopathy	57
Thin corpus callosum	3
Other lesions	4
Other findings	7
Not considered	5
**Causes – genetic alterations (out of those sequenced)**
*SCN1A*	2/32
*KCNT1*	10/30
*TBC1D24*	2/15
Not considered	47

Patients diagnosed with MMPSI are unlikely to achieve intellectual growth, learning, and other developmental milestones. Following an early onset, MMPSI seizures increase in frequency to the point of halting normal development; patients also lose any developmental progress they had previously accomplished (Coppola et al., 1995). Even when the seizures diminish in frequency, very few patients resume neurodevelopmental growth. The end results are severe delays in development and profound intellectual disability. Not surprisingly, absence of language and hypotonia are also commonly noted in these patients. In capturing the bleak prognosis for MMPSI patients, a study of 14 patients concluded, “the highest developmental level maintained beyond 1 year of age in all patients was partial head control, rolling and visual fixation” (McTague et al., 2013). Out of the 96 patients considered in **Table 2**, 20 were reported as deceased.

Possible cause(s) for seizure development in MMPSI patients has remained elusive until recently. Neurometabolic, blood gas and serum tests are typically normal, and brain lesions are rarely observed in affected patients (Nabbout and Dulac, 2008). Besides microcephaly, or abnormally small heads, that progressively appeared in 57 out of 91 examined patients reported in the literature (**Table 2**), the brain appears to be without any other structural lesions at presentation.

Genetic etiologies for MMPSI were first made in 2011, with the discovery of *SCN1A* (Nav1.1) mutations (Carranza Rojo et al., 2011), followed by *TBC1D24* (protein name the same; Milh et al., 2013) and *KCNT1* (Slack) mutations (Barcia et al., 2012; Epi et al., 2013; Ishii et al., 2013; McTague et al., 2013; Vanderver et al., 2014). Of special note, Slack channel mutations have been found in 50% of patients examined (Barcia et al., 2012). Detailed characterizations of these Slack mutations are presented at the end of this section.

### AUTOSOMAL DOMINANT NOCTURNAL FRONTAL LOBE EPILEPSY

Autosomal dominant nocturnal frontal lobe epilepsy is a focal seizure that occurs predominantly during sleep with a typical onset in late childhood. The mean age among more than 110 patients reported in six different case reports was 10.9 years (**Table 3**; Scheffer et al., 1995; Oldani et al., 1998; Phillips et al., 1998; Nakken et al., 1999; Derry et al., 2008; Heron et al., 2012). ADNFLE patients are sometimes misdiagnosed as having sleep disorders rather than suffering a seizure attack, because the seizure attacks often disrupt sound sleep (Oldani et al., 1998).

**Table 3:** Summary of clinical reports on ADNFLE patients.

No. of patients reviewed	114
No. of Refs. reviewed	6
Males	48%
**Age at onset**
Average	10.9 year
Range	1–30 year
Deceased	N/A

That a genetic mutation can result in ADNFLE in an affected family was first suggested by chromosome linkage and confirmed later by sequencing of the gene for the nicotinic α4 acetylcholine receptor subunit (*CHRNA4*; Steinlein et al., 1995). For this reason, ADNFLE is most commonly associated with mutations in acetylcholine receptor subunits (Steinlein et al., 1995; De Fusco et al., 2000; Aridon et al., 2006). Statistically, however, only 20% of ADNFLE patients with a family history of seizures, and 5% of those without, have a mutation in one of these genes (Kurahashi and Hirose, 2002).

More recently, mutations in *KCNT1* (Slack) have been identified as a novel genetic etiology for ADNFLE, but these too seem to be a cause in a minority of affected families (Heron et al., 2012). Nevertheless, it is interesting that several observations distinguish the families harboring a *KCNT1* mutation from those with a different mutation. As a notable example, the occurrence of intellectual disability and other psychiatric illnesses appear to be greatly increased in those families with a *KCNT1* mutation (Heron et al., 2012). This is in contrast to ADNFLE patients without mutations in Slack, in whom intelligence and other neurologic functions are largely unimpaired (Phillips et al., 1998). Penetrance of the mutation is also increased to 100% in the families with Slack mutations, when that of acetylcholine receptor mutations has been estimated to be only 70% (Heron et al., 2012). These results further implicate Slack channels in intellectual development.

### OHTAHARA SYNDROME

Originally described as an early infantile epileptic encephalopathy with suppression-bursts (Ohtahara et al., 1976), OS is one of the earliest seizures in its presentation (Clarke et al., 1987). Among 82 patients reported in 27 different publications (Robain and Dulac, 1992; Miller et al., 1998; Ohno et al., 2000; Ohtsuka et al., 2000; Krasemann et al., 2001; Quan et al., 2001; Trinka et al., 2001; Krsek et al., 2002; Yamatogi and Ohtahara, 2002; Hmaimess et al., 2005; Kato et al., 2007, 2010, 2013; Saitsu et al., 2008, 2010, 2012a,b; Absoud et al., 2010; Cazorla et al., 2010; Fullston et al., 2010; Giordano et al., 2010; Seo et al., 2010; Choi et al., 2011; Eksioglu et al., 2011; Milh et al., 2011; Nakamura et al., 2013; Touma et al., 2013), the first seizure was seen within 3 weeks of life (**Table 4**). Common prognosis and known etiologies of OS are summarized below.

**Table 4:** Summary of clinical reports on OS patients.

No. of patients reviewed	82
No. of Refs. reviewed	27
Males	60%
**Age at onset**
Average	2.56 week
Range	1 day – 4 month
**Prognosis**
Evolution to WS	43%
Developmental delay	83%
Normal development	10%
Hypotonia	52%
Deceased	22%
**Causes – brain abnormalities (out of those considered)**
Normal	17/67
Atrophy	28/67
Delayed myelination	13/67
Encephalopathy	8/67
Thin corpus callosum	9/67
Other lesions	9/67
Other findings	13/67
Not considered	15
**Causes – genetic alterations (out of those sequenced)**
*SCN2A*	12/25
*KCNT1*	3/25
*KCNQ2*	13/25
*STXBP1*	20/45
Other genes	8/45
Not considered	37

A majority of OS patients show severe developmental delay, including intellectual disability. Greater than 80% of OS patients reported in the literature have a developmental delay, while only 10% are described as showing normal development (**Table 4**). OS patients also appear to have increased vulnerability to other ailments such as pneumonia and virus infections (Krasemann et al., 2001; Quan et al., 2001), and these complications have been a cause of death in more than 20% of patients. It remains a challenge to reverse or overcome these prognoses, since these seizures have pronounced pharmacological resistance (Beal et al., 2012). Nevertheless, surgical intervention may hold some promise for patients in whom brain abnormalities can be identified as the basis of the seizures (Malik et al., 2013). Macroscopic and microscopic brain abnormalities are the predominant causes of seizure development in OS patients, and common defects are enumerated in **Table 4** (Robain and Dulac, 1992; Trinka et al., 2001; Low et al., 2007; Saitsu et al., 2008; Nakamura et al., 2013). In more than one-fifth of the patients, however, no brain abnormalities can be detected.

Genetic etiologies have also been identified in a subset of OS patients. To date, alterations in five different genes have been found in patients: ion channels *KCNQ2* (Kv7.2; Saitsu et al., 2012a; Kato et al., 2013), *SCN2A* (Nav1.2; Nakamura et al., 2013; Touma et al., 2013), and *KCNT1* (Slack; Martin et al., 2014); the transcription factor ARX (Kato et al., 2007; Absoud et al., 2010; Giordano et al., 2010; Fullston et al., 2010; Eksioglu et al., 2011); and the synaptic binding protein *STXBP1* (Saitsu et al., 2008, 2010, 2011; Mignot et al., 2011; Milh et al., 2011). Interestingly, mutations in *SCN2A* and *KCNT1* have also been found in patients diagnosed with MMPSI.

Many earlier studies (prior to 2011) were selective in their approach, sequencing only one or a few selected genes of interest. A growing number of researchers are now utilizing whole exomic or genomic sequencing for such patients (Majewski et al., 2011), however, and it is foreseeable that a more comprehensive estimate of the prevalence of these epileptogenic alleles will emerge within the next decade.

## MECHANISMS UNDERLYING CHANGES IN HUMAN SLACK MUTANTS

Slack mutants have been tested for change in channel activity in *Xenopus laevis* oocytes and HEK 293 cells using two-electrode voltage clamping. These studies have shown that, surprisingly, currents generated by the Slack mutants are greatly increased over those in wild type channels. Peak current amplitudes of mutant Slack currents are increased by 3- to 12-fold, with no change in levels of Slack protein (Barcia et al., 2012; Martin et al., 2014; Milligan et al., 2014).

One alteration in the biophysical properties of the mutant Slack channels is that the occurrence of subconductance states is greatly reduced compared to that in wild type channels. As we described earlier, subconductances appear as brief, flickering short steps alternating with time spent in the fully open or the closed state in single channel patch clamp experiments. The wild type channel spends most of its time transitioning between the closed or subconductance states (Joiner et al., 1998). However, mutant channels are more likely to open immediately to a fully open state rather than to a subconductance state, resulting in an overall increase in current during depolarization of the membrane (Barcia et al., 2012). A similar reduction in occurrence of subconductance states was also seen in FMRP-mediated positive regulation of Slack channel activity (Brown et al., 2010).

A second mechanism for increased current in at least two of the mutant channels is that they render the channels in a state that mimics constitutive channel phosphorylation by protein kinase C (Barcia et al., 2012). Wild type Slack channels undergo phosphorylation by this enzyme at a site (Serine 407) in their large C-terminal cytoplasmic domain, leading to an ∼3-fold increase in peak current amplitude. Protein kinase C was pharmacologically activated in *Xenopus* oocytes expressing Slack channels, and the peak current amplitude compared in the mutants and wild type channels using two-electrode voltage clamping. The results showed that unlike in the wild type channel, which showed an increase in channel activity, it remained unchanged in mutant channels (Barcia et al., 2012). Thus in these channels the mutations both mimicked and occluded the effects of activation by protein kinase C.

Other mechanisms for the enhanced currents in the Slack mutants are under investigation. These channels are sensitive to cytoplasmic levels of Na^+^. In patch clamp experiments it has been found, however, that the Na^+^-sensitivity of the mutant channels is not different from that of the wild type channels. Nevertheless, other potential mechanisms, such as shifts in voltage-dependence, may also contribute to the enhanced currents in mutant channels.

The unexpected finding that a gain-of-function change in a K^+^ channel can induce a hyperexcitable state of the brain has a precedence in the BK channel, a mutation of which can lead to generalized epilepsy and paroxysmal dyskinesia (GEPD; Yang et al., 2010). BK channels are activated by [Ca^2+^]_i_, and can contribute to the rapid hyperpolarizations that follow action potentials, thereby regulating cellular excitability. An electrophysiological study of the mutant BK channel in *Xenopus* oocytes showed that the mutant channel has increased Ca^2+^ sensitivity, resulting in an overall increase in BK channel activity (Yang et al., 2010).

Several possible changes at the cellular/neuronal network level could account for how aberrant electrical activities of the brain may arise from increased K^+^ channel activity, some of which have been suggested by others (Du et al., 2005). First, an increase in K^+^ current could cause more rapid neuronal repolarization, shortening the duration of action potentials. A more rapid repolarization can indirectly increase cell excitability by increasing the rate at which voltage-dependent Na^+^ channels recover from inactivation. Next, more pronounced hyperpolarizations resulting from BK or Slack channel hyperactivity may also potentiate hyperpolarization-activated cation channel (*I*_h_) currents, aberrantly triggering network excitability. It is also possible that the enhancement of K^+^ current may occur selectively in inhibitory neurons. This could lead to a selective suppression of the activity of inhibitory interneurons, thereby producing an imbalance of excitation to inhibition (Du et al., 2005). Finally, increases in K^+^ current early in development could alter the formation of normal patterns of synaptic connections, predisposing the nervous system to develop circuits that generate epileptiform discharges. After close to a decade, however, these hypotheses have yet to be tested experimentally, perhaps due in part because of a lack of a specific knock-in mouse or other animal models.

Malignant migrating partial seizures of infancy, OS, and ADNFLE have traditionally been regarded as distinct seizures with considerable heterogeneity in etiology and prognosis. OS and MMPSI are two of the first epilepsies known to affect newborns, and both produce devastating changes in neurodevelopment. ADNFLE, on the other hand, is typically less disruptive of normal development and life, and seizures are often successfully controlled with antiepileptic drugs. The recent discoveries that Slack mutants have been uncovered in patients diagnosed with one of these three seizures, and that they all share in common severe intellectual disability and other developmental delays together give rise to an emerging role for Slack channels in intellectual disability. The evidence suggests that a key physiological role of Slack may be its control over cellular or network excitability in regions of the brain involved in intellectual development.

## CONCLUSION

Slack channels are physiologically important regulators of neuronal excitability and adaptability to changing patterns of sensory stimulation. In this review, we have considered how alterations in Slack channel activity can have pathophysiological ramifications, in conditions such as FXS and early onset epileptic encephalopathies. In addition to FXS, which has a well-established genetic link to the development of intellectual disability, the three seizures related to Slack mutants – OS, MMPSI and ADNFLE – also notably share a common manifestation of intellectual disability in their patients. These new findings make a strong argument that Slack channels may be a common link that can describe the occurrence of intellectual disability in these patients, suggesting that Slack channels could be critical modulators of cognitive development.

## Conflict of Interest Statement

The authors declare that the research was conducted in the absence of any commercial or financial relationships that could be construed as a potential conflict of interest.
